# Selective Exposure of Robust Perovskite Layer of Aurivillius‐Type Compounds for Stable Photocatalytic Overall Water Splitting

**DOI:** 10.1002/advs.202302206

**Published:** 2023-05-31

**Authors:** Jie Huang, Yuyang Kang, Jian‐An Liu, Ruotian Chen, Tengfeng Xie, Zhongran Liu, Xiaoxiang Xu, He Tian, Lichang Yin, Fengtao Fan, Lianzhou Wang, Gang Liu

**Affiliations:** ^1^ Shenyang National Laboratory for Materials Science Institute of Metal Research Chinese Academy of Sciences 72 Wenhua Road Shenyang 110016 China; ^2^ School of Materials Science and Engineering University of Science and Technology of China 72 Wenhua Road Shenyang 110016 China; ^3^ State Key Laboratory of Catalysis Dalian National Laboratory for Clean Energy iChEM Dalian Institute of Chemical Physics Chinese Academy of Sciences Dalian 116023 China; ^4^ College of Chemistry Jilin University Changchun 130012 China; ^5^ Center of Electron Microscopy School of Materials Science and Engineering Zhejiang University Hangzhou 310027 China; ^6^ School of Chemical Science and Engineering Tongji University Shanghai 200092 China; ^7^ Nanomaterials Centre School of Chemical Engineering and Australian Institute for Bioengineering and Nanotechnology The University of Queensland St Lucia QLD 4072 Australia

**Keywords:** Aurivillius compounds, overall water splitting, perovskites, photocatalysis, surface termination

## Abstract

Aurivillius‐type compounds ((Bi_2_O_2_)^2+^(A_n__–1_B_n_O_3__n__+1_)^2−^) with alternately stacked layers of bismuth oxide (Bi_2_O_2_)^2+^ and perovskite (A_n__−1_B_n_O_3__n__+1_)^2−^ are promising photocatalysts for overall water splitting due to their suitable band structures and adjustable layered characteristics. However, the self‐reduction of Bi^3+^ at the top (Bi_2_O_2_)^2+^ layers induced by photogenerated electrons during photocatalytic processes causes inactivation of the compounds as photocatalysts. Here, using Bi_3_TiNbO_9_ as a model photocatalyst, its surface termination is modulated by acid etching, which well suppresses the self‐corrosion phenomenon. A combination of comprehensive experimental investigations together with theoretical calculations reveals the transition of the material surface from the self‐reduction‐sensitive (Bi_2_O_2_)^2+^ layer to the robust (BiTiNbO_7_)^2−^ perovskite layer, enabling effective electron transfer through surface trapping and effective hole transfer through surface electric field, and also efficient transfer of the electrons to the cocatalyst for greatly enhanced photocatalytic overall water splitting. Moreover, this facile modification strategy can be readily extended to other Aurivillius compounds (e.g., SrBi_2_Nb_2_O_9_, Bi_4_Ti_3_O_12_, and SrBi_4_Ti_4_O_15_) and therefore justify its usefulness in rationally tailoring surface structures of layered photocatalysts for high photocatalytic overall water‐splitting activity and stability.

## Graphical abstract

By rationally tailoring the surface structure of Aurivillius compounds to expose robust perovskite layer, stable photocatalytic overall water splitting with greatly enhanced activity is achieved by both inhibiting the Bi^3+^ self‐reduction and promoting the effective migration of photogenerated electrons to the cocatalyst.

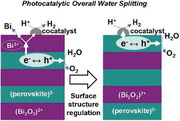

## 1 Introduction

Photocatalytic overall water splitting is one of the most effective ways to convert solar energy into chemical energy.^[^ ^1^ ^]^ Although photocatalytic technique opens up a promising scenario to set up a clean and sustainable energy infrastructure, it awaits significant breakthroughs to promote practical applications, given the low solar to hydrogen energy conversion efficiency and/or also low stability in some cases. Among various photocatalytic systems, Aurivillius‐type compounds are of particular interest for promoting charge carrier separation due to structural distortion caused by interlayer interaction.^[^ ^2^ ^]^ Currently, some Aurivillius compounds such as Bi_4_Ti_3_O_12_, Bi_3_TiNbO_9_, PbBi_2_Nb_2_O_9_ exhibit efficient photocatalytic hydrogen or oxygen evolution activity from water splitting in the presence of sacrificial agent and PbBi_2_Nb_2_O_9_ even shows photocatalytic activity under visible light.^[^ ^3^ ^]^ However, the Pourbaix diagram of the Bi‐H_2_O system suggests that Bi^3+^ intrinsically tends to transform into metallic Bi at the potential of proton reduction to hydrogen, so that bismuth‐based compounds are prone to self‐reduction with regard to unfavorable photocatalytic water splitting.^[^ ^4^ ^]^ This self‐reduction phenomenon has been experimentally observed by the appearance of a reduction peak of Bi^3+^ in bismuth oxide materials under an applied bias potential required for electrocatalytic reduction of carbon dioxide.^[^ ^5^ ^]^ A similar process also occurs in photocatalysis.^[^ ^6^ ^]^ Although various Bi‐based photocatalysts have been developed, few solutions have been proposed to address the instability issue involved in photocatalytic processes. The resulting metallic Bi from Bi^3+^ reduction can act as carrier quenching center and also lead to the shedding of cocatalyst, which greatly impair the water‐splitting process and induce further self‐reduction.^[^ ^7^ ^]^ There is thereby an urgent need to develop effective strategies to suppress the self‐reduction of Aurivillius compounds for stable photocatalytic overall water splitting.

Crystal‐facet‐dependent stability and activity have been noticed in a wide range of faceted photocatalysts with strong isotropic bonds because surface atomic arrangements and electronic structures vary sensitively with exposed facets.^[^ ^8^ ^]^ However, the application of selective facet exposure in layered materials with anisotropic bonds seems difficult because their particle surface usually consists of only two kinds of facets with diverse properties, e.g., the basal {001} surface and the lateral surface parallel to the direction of layered stacking.^[^ ^9^ ^]^ The effective masses of photogenerated electrons and holes differ greatly in different directions, resulting in electrons tending to migrate along the c axis to the {001} facet, while holes are confined within the layer and tend to migrate to the {110} facet.^[^ ^3^, ^10^ ^]^ One fascinating strategy is to selectively expose the desirable layer as a surface terminal layer to modulate the behavior of photogenerated charge carriers. Inspired by the previous theoretical results that the conduction band minimum (CBM) and valence band maximum (VBM) of Aurivillius compound Bi_3_TiNbO_9_ are dominantly contributed by its perovskite (BiTiNbO_7_)^2−^ blocks,^[^ ^11^ ^]^ selective exposure of the perovskite layer as a photocatalytic active unit is anticipated to favor not only the transfer of photocarriers but suppression of self‐reduction of the (Bi_2_O_2_)^2+^ layer under photocatalytic water‐splitting conditions.

In this study, Bi_3_TiNbO_9_ was chosen as a model photocatalyst to develop the above proposed proof‐of‐concept for stable overall water splitting. The surface terminal layer of Bi_3_TiNbO_9_ nanosheets can be well controlled by the combination of molten salt synthesis and subsequent acid etching processes, according to experimental and theoretical investigations. It was revealed that Bi_3_TiNbO_9_ with robust (BiTiNbO_7_)^2−^ layer termination (denoted Bi_3_TiNbO_9_‐PL) can lead to much superior photocatalytic overall water‐splitting activity and stability, while Bi_3_TiNbO_9_ with (Bi_2_O_2_)^2+^ layer termination (denoted Bi_3_TiNbO_9_‐BL) causes both low activity and poor stability under the same conditions. Moreover, this straightforward strategy can be readily extended to other Aurivillius compounds (e.g., SrBi_2_Nb_2_O_9_, Bi_4_Ti_3_O_12_, and SrBi_4_Ti_4_O_15_) and therefore justify its usefulness in rationally tailoring the surface structures of layered photocatalysts for high photocatalytic activity and stability.

## 2 Results and Discussion

### 2.1 Surface Structure Differences between Bi_3_TiNbO_9_‐BL and Bi_3_TiNbO_9_‐PL

**Figure 1:**
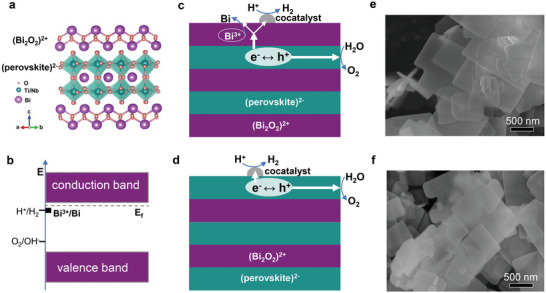
Relationship between surface structure and photocatalytic stability. Schematic of a) crystal structure of Bi_3_TiNbO_9_ and b) its band structure referring to the potentials for water splitting and Bi^3+^ reduction. Schematic for photocatalytic water‐splitting processes over cocatalyst‐modified Bi_3_TiNbO_9_ with surface termination of c) (Bi_2_O_2_)^2+^ layer and d) (BiTiNbO_7_)^2−^ layer. Scanning electron microscopy (SEM) images of e) Bi_3_TiNbO_9_‐BL and f) Bi_3_TiNbO_9_‐PL.

Bi_3_TiNbO_9_ is a layered compound consisting of an alternate (Bi_2_O_2_)^2+^ layer and (BiTiNbO_7_)^2−^ layer along the c axis, as shown in **Figure** **1**a. Although the (Bi_2_O_2_)^2+^ layer is the typical building block for Aurivillius‐type compounds, it is the (BiTiNbO_7_)^2−^ perovskite layer that determines the band structures. Bi_3_TiNbO_9_ has its CBM and VBM straddling hydrogen and oxygen evolution potentials, warranting promising applications for photocatalytic overall water splitting (Figure 1b). However, the easily reduced characteristic of Bi^3+^ ions ($\varphi_{{\mathrm{Bi}}^{3+}/\mathrm{Bi}}$ close to $\varphi_{H^{+}/H_{2}}$) has a strong implication that charge transfer ability becomes a key factor affecting the stability of materials.^[^ ^12^ ^]^ In the case of Bi_3_TiNbO_9_, whose surface is terminated by the (Bi_2_O_2_)^2+^ layer (Bi_3_TiNbO_9_‐BL), photogenerated electrons have to migrate across the insulating (Bi_2_O_2_)^2+^ layer^[^ ^13^ ^]^ and transfer to the cocatalyst to participate in the hydrogen evolution reaction (Figure 1c). The complex charge transfer process results in the reduction of Bi^3+^ ions at the interface between photocatalytic material and cocatalyst to metallic Bi with low work function^[^ ^14^ ^]^ and weak activity.^[^ ^15^ ^]^ Consequently, photogenerated electrons tend to accumulate in metallic Bi and cannot be consumed by protons timely, leading to further corrosion of the (Bi_2_O_2_)^2+^ layer and shedding of the cocatalyst. On the contrary, electrons can be transferred directly to the cocatalyst for photocatalytic hydrogen evolution reaction when Bi_3_TiNbO_9_ has its surface terminated by a robust perovskite (BiTiNbO_7_)^2−^ layer (Bi_3_TiNbO_9_‐PL) (Figure 1d). This not only effectively reduces the carrier migration distance but also ensures stable adhesion of the cocatalyst to the perovskite layer. From these considerations, surface regulation is highly desirable for Bi_3_TiNbO_9_ to achieve stable high photocatalytic overall water‐splitting activity.

The difference in bonding strength within Bi_3_TiNbO_9_ generally leads to the exposure of {001} crystal facet which has a low surface energy.^[^ ^3^, ^16^ ^]^ Moreover, the Cl^−^ ions in the molten salt have a strong electrostatic interaction with the (Bi_2_O_2_)^2+^ layer, which not only hinders the preferential growth of the material along the c‐axis but also promotes the surface to be terminated by (Bi_2_O_2_)^2+^ layer. Thereby, the molten salt‐based synthetic route used in this study favors the formation of Bi_3_TiNbO_9_ with (Bi_2_O_2_)^2+^ layer termination (Bi_3_TiNbO_9_‐BL) (Figure 1c). Because Bi—O bonds are weaker than Ti(Nb)—O bonds,^[^ ^17^ ^]^ it is feasible to dissolve Bi^3+^ ions in the surface layer by using hydrochloric acid while leaving the perovskite layer intact. Moreover, the (Bi_2_O_2_)^2+^ layers in the bulk are protected by perovskite layers, which allow selective etching of the top (Bi_2_O_2_)^2+^ layers to produce Bi_3_TiNbO_9_ with (BiTiNbO_7_)^2−^ layer termination (Bi_3_TiNbO_9_‐PL) (Figure 1d). The resulting Bi_3_TiNbO_9_‐PL sample has a very similar morphology to Bi_3_TiNbO_9_‐BL (Figure 1e,f) and both samples share nearly the same X‐ray diffraction patterns (Figure S1, Supporting Information), confirming the good retaining of crystal structure of Bi_3_TiNbO_9_ after acid etching. The band structure of the two samples was further analyzed from their UV‐vis diffuse reflectance spectra and Mott–Schottky curves in Figure S2 in the Supporting Information. Both Bi_3_TiNbO_9_‐BL and Bi_3_TiNbO_9_‐PL exhibit the bandgap of 3.23 eV and have the position of conduction and valence bands at −0.87 and 2.36 V versus reversible hydrogen electrode (RHE), respectively. It is worth noting that Bi_3_TiNbO_9_‐PL has a stronger absorption intensity in the range of 400–700 nm than Bi_3_TiNbO_9_‐BL, which could be caused by surface states.^[^ ^18^ ^]^

**Figure 2:**
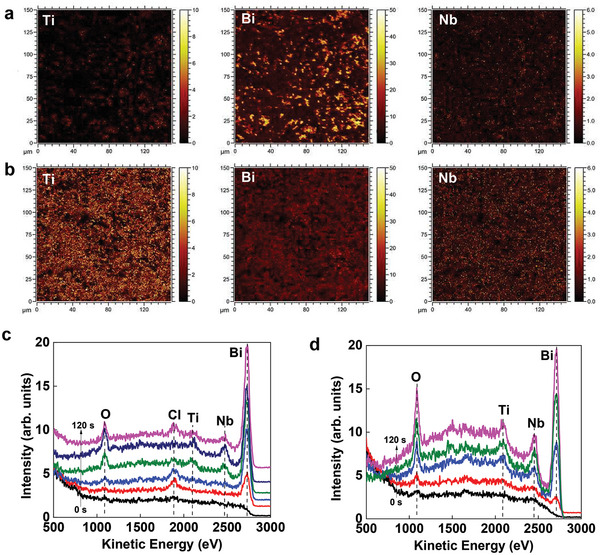
Macroscopic surface structure verification. TOF‐SIMS images of Ti, Bi, and Nb for a) Bi_3_TiNbO_9_‐BL and b) Bi_3_TiNbO_9_‐PL. High‐sensitivity low‐energy ion scattering spectra for c) Bi_3_TiNbO_9_‐BL and d) Bi_3_TiNbO_9_‐PL.

The distribution of Bi, Ti, and Nb compositions at the surface layer of Bi_3_TiNbO_9_‐BL and Bi_3_TiNbO_9_‐PL was first analyzed by time‐of‐flight secondary ion mass spectrometry (TOF‐SIMS), where the element content at the surface can be evaluated by the brightness of the element signal image. The TOF‐SIMS images in **Figure** **2**a,b show that Bi_3_TiNbO_9_‐BL has much more Bi composition than Bi_3_TiNbO_9_‐PL in their surface layers, while Ti(Nb) is in the opposite trend. Inductively coupled plasma‐atomic emission spectrometry (ICP‐OES) analysis confirms that the acid solution after etching Bi_3_TiNbO_9_‐BL contains a high concentration of Bi^3+^ ions (Table S1, Supporting Information). A control experiment using deionized water rather than hydrochloric acid gave no detectable ICP‐OES signal for Bi^3+^ ions. The etching is mostly limited to the surface layer of Bi_3_TiNbO_9_ as revealed by the combined ICP‐OES and X‐ray photoelectron spectroscopy (XPS) investigations (Figure S3, Supporting Information), which show a 42% reduction in Bi on the surface but only a 5.5% reduction in bulk. The absence of detectable signal by electron spin resonance spectroscopy (Figure S4, Supporting Information) suggests that acid etching removes the entire (Bi_2_O_2_)^2+^ layer and leads to no paramagnetic species such as Bi vacancies. In addition, compared to Bi_3_TiNbO_9_‐BL, Bi_3_TiNbO_9_‐PL has much better hydrophilicity property as indicated by the contact angle decrease from 49.75° to 16.125° (Figure S5, Supporting Information) and the positive shift in binding energy from 206.3 to 206.48 eV for Nb 3d_5/2_ (Figure S6, Supporting Information).

The surface structure of both Bi_3_TiNbO_9_ samples was further studied by high‐sensitivity low‐energy ion scattering spectroscopy (HS‐LEIS), which owns a detecting depth of only two atomic layers. Figure 2c,d shows statistical signals of surface elements at different sputtering times of He^+^ ions. At the initial stage, no signal appears from their pristine surfaces due to surface adsorption saturation caused by exposure to air. After He^+^ ion cleaning, weak Bi and O signals immediately emerge for Bi_3_TiNbO_9_‐BL rather than Ti(Nb) signal. On the contrary, Bi, O, and Ti(Nb) signals can be detected simultaneously for Bi_3_TiNbO_9_‐PL, which is characteristic of the perovskite (BiTiNbO_7_)^2−^ layer. These results further confirm that Bi_3_TiNbO_9_‐BL and Bi_3_TiNbO_9_‐PL have different surface structures, i.e., a (Bi_2_O_2_)^2+^ type surface and a (BiTiNbO_7_)^2−^ type one, respectively. With extended He^+^ ion sputtering time, Ti(Nb) signals gradually appear for Bi_3_TiNbO_9_‐BL, indicating that the perovskite (BiTiNbO_7_)^2−^ layer is underneath the (Bi_2_O_2_)^2+^ layer. It is noteworthy that only Bi_3_TiNbO_9_‐BL contains the Cl signal due to the residual Cl^−^ ions from the molten salt that strongly bonded to the top (Bi_2_O_2_)^2+^ layers of Bi_3_TiNbO_9_‐BL.

**Figure 3:**
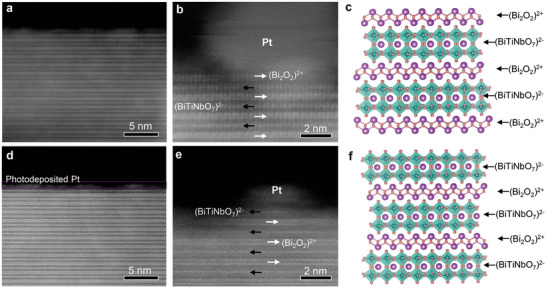
Atomic structure analysis of surface terminations. Atomic‐level cross‐section HAADF‐STEM images and corresponding atomic structure schematics for a–c) Bi_3_TiNbO_9_‐BL and d–f) Bi_3_TiNbO_9_‐PL, where purple and green balls represent Bi atoms and Ti(Nb) atoms, respectively.

For direct visualization of the surface termination, Bi_3_TiNbO_9_ samples with photodeposited Pt cocatalyst were cut by ultrathin sectioning and the cross‐section was inspected by high angle annular dark field scanning transmission electron microscopy (HAADF‐STEM). In the high angle detection mode, the image contrast is proportional to the square of the atomic number, which enables easy discrimination of different Bi‐containing layers according to their bright spots in **Figure** **3**. In this case, the bilayer bright spots represent the (Bi_2_O_2_)^2+^ layer and the monolayer bright spots represent the (BiTiNbO_7_)^2−^ layer. It is evident that the (Bi_2_O_2_)^2+^ and (BiTiNbO_7_)^2−^ layers are stacked alternately, and the thickness of the two alternate layers is estimated to be 1.259 nm. Apparently, the Bi_3_TiNbO_9_‐BL surface is terminated by a (Bi_2_O_2_)^2+^ layer while the Bi_3_TiNbO_9_‐PL surface is terminated by a (BiTiNbO_7_)^2−^ layer. In addition, the Pt cocatalyst is in contact with the surface layers of two Bi_3_TiNbO_9_ samples, presumably via Bi—O—Pt and Ti(Nb)—O—Pt bonding for Bi_3_TiNbO_9_‐BL and Bi_3_TiNbO_9_‐PL, respectively. The interface structure plays a decisive role in controlling charge transfer, which thus determines the activity and stability of the photocatalyst.

### 2.2 Photocatalytic Properties of Bi_3_TiNbO_9_‐BL and Bi_3_TiNbO_9_‐PL

**Figure 4:**
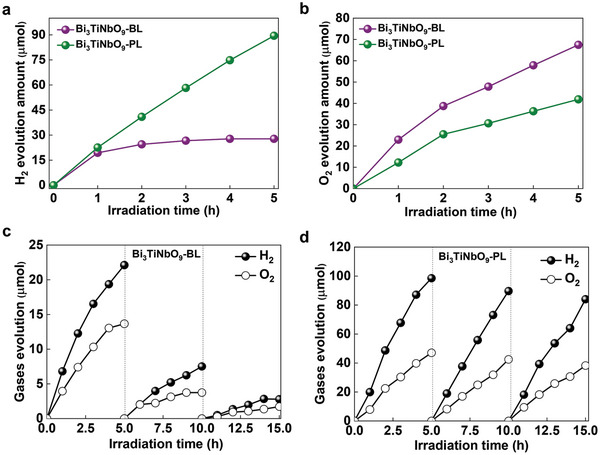
Activity and stability of photocatalytic water splitting. Comparison of half‐reactions in photocatalytic water splitting: a) hydrogen evolution (reaction conditions: 50 mg photocatalyst with 1 wt% Pt cocatalyst, 10 mL methanol + 90 mL H_2_O), b) oxygen evolution (reaction conditions: 50 mg photocatalyst, 850 mg AgNO_3_, 100 mL H_2_O). Time courses of photocatalytic overall water splitting over c) Bi_3_TiNbO_9_‐BL and d) Bi_3_TiNbO_9_‐PL (reaction conditions: 50 mg photocatalyst, 0.6 wt% Rh/Cr_2_O_3_ cocatalyst, 100 mL H_2_O).

The photocatalytic performance and stability were first investigated by hydrogen evolution and oxygen evolution reactions in the presence of sacrificial agents. For photocatalytic hydrogen evolution, methanol was used to scavenge photogenerated holes and photogenerated electrons participate in the water reduction reaction. **Figure** **4**a compares the time course of hydrogen evolution for Bi_3_TiNbO_9_‐BL and Bi_3_TiNbO_9_‐PL with the photodeposited Pt cocatalyst. Although both samples show similar photocatalytic activity at the initial stage, they exhibit diverse performance under extended irradiation time. Specifically, the activity of Bi_3_TiNbO_9_‐BL quickly degraded and became almost inactive after 2 h irradiation. This is in sharp contrast to Bi_3_TiNbO_9_‐PL, which maintains a much higher activity and stability for the whole 5 h irradiation. When AgNO_3_ was used as the sacrificial agent for the oxygen evolution reaction, both samples showed continuous oxygen evolution under light irradiation and the performance order was reversed (Figure 4b). The weak activity decline with irradiation time can be attributed to the adhesion of reduced metallic Ag on the surface of Bi_3_TiNbO_9_, which partially blocks light penetration. From the results of hydrogen and oxygen evolutions, it is reasonably speculated that Bi_3_TiNbO_9_‐BL is subjected to self‐corrosion via the reduction of Bi^3+^ in the upmost (Bi_2_O_2_)^2+^ layer, which can be suppressed by modifying the surface structures to expose robust (BiTiNbO_7_)^2−^ perovskite layer.

To further evaluate the photocatalytic performance, the appropriate cocatalyst for photocatalytic overall water splitting reactions was deposited on Bi_3_TiNbO_9_. Rh/Cr_2_O_3_ core–shell cocatalyst is known to promote overall water‐splitting reactions without reverse reaction.^[^ ^19^ ^]^ Therefore, we deposited Rh/Cr_2_O_3_ core–shell cocatalyst onto Bi_3_TiNbO_9_ by two‐step photo‐deposition processes. The photocatalytic overall water‐splitting performance of two Bi_3_TiNbO_9_ samples in Figure 4c,d shows that although hydrogen and oxygen evolution were realized with Bi_3_TiNbO_9_‐BL, the activity decayed rapidly during the long‐term test. Moreover, the molar ratio of hydrogen to oxygen evolved in the initial stage was less than 2:1, probably because some photogenerated electrons were consumed by the Bi^3+^ reduction reactions. On the contrary, Bi_3_TiNbO_9_‐PL gave a much higher activity for photocatalytic overall water splitting for three consecutive cycle tests (Figure 4d), indicative of a much superior stability. The gas evolution rate reached 21.78 µmol h^−1^ 50 mg^−1^ for H_2_ and 9.94 µmol h^−1^ 50 mg^−1^ for O_2_, which is 4.5 times higher than that of Bi_3_TiNbO_9_‐BL. In addition, Bi_3_TiNbO_9_‐PL achieved an apparent quantum yield (AQY) of 0.26% at 365 nm, which is greatly improved compared with that of Bi_3_TiNbO_9_‐BL (AQY = 0.068%). Figure S7 in the Supporting Information shows the UV‐vis diffuse reflectance spectra of Bi_3_TiNbO_9_‐Rh/Cr_2_O_3_ before and after photocatalytic overall water splitting. Absorption below 400 nm contributes to the intrinsic bandgap absorption of Bi_3_TiNbO_9_, while strong background absorption in the range of 400–700 nm is ascribed to localized surface plasmon resonance or interband transition of Rh cocatalyst deposited on Bi_3_TiNbO_9_.^[^ ^20^ ^]^ The background absorption of Bi_3_TiNbO_9_‐BL‐cocatalyst is significantly stronger than that of Bi_3_TiNbO_9_‐PL‐cocatalyst largely because the metallic Bi was produced during the photodeposition process of cocatalyst. The results are consistent with the sample color after cocatalyst deposition (Figure S8, Supporting Information), where Bi_3_TiNbO_9_‐BL‐cocatalyst is apparently darker than Bi_3_TiNbO_9_‐PL‐cocatalyst. Nevertheless, the background absorption intensity of Bi_3_TiNbO_9_‐BL decreased much after the test. This is probably due to the detachment of cocatalyst from Bi_3_TiNbO_9_‐BL sheets during the reaction process. In contrast, the background absorption of Bi_3_TiNbO_9_‐PL remained almost unchanged after the test, indicating the good stability of the photocatalyst. Therefore, by modifying the surface termination of Bi_3_TiNbO_9_, the photocorrosion problem was solved to induce stable and high photocatalytic overall water splitting.

The photocorrosion phenomenon is common for this kind of Aurivillius compound. The effectiveness of our strategy of selectively exposing corresponding perovskite layers has been further validated in other Aurivillius compounds. For instance, the surface terminations of three typical Aurivillius compounds (SrBi_2_Nb_2_O_9_, Bi_4_Ti_3_O_12_, and SrBi_4_Ti_4_O_15_) were changed with the same acid etching. Figures S9–S11 in the Supporting Information show X‐ray diffraction patterns of Aurivillius compounds terminated with the (Bi_2_O_2_)^2+^ layer (SrBi_2_Nb_2_O_9_‐BL, Bi_4_Ti_3_O_12_‐BL, and SrBi_4_Ti_4_O_15_‐BL) and perovskite layer (SrBi_2_Nb_2_O_9_‐PL, Bi_4_Ti_3_O_12_‐PL, and SrBi_4_Ti_4_O_15_‐PL). Their patterns are basically the same, indicating the retaining of bulk crystal structure after acid etching. Being consistent with the results of Bi_3_TiNbO_9_, all three Aurivillius compounds with perovskite terminated surfaces deliver much more stable photocatalytic hydrogen evolution than their counterparts with (Bi_2_O_2_)^2+^ terminated surfaces (Figures S12–S14, Supporting Information). Comparison of the photographs of SrBi_2_Nb_2_O_9_‐BL/PL suspensions after the reaction (Figures S15, Supporting Information) clearly shows the darker color of the former with the (Bi_2_O_2_)^2+^ terminated surface, arising from the formed metallic Bi precipitation.

### 2.3 Mechanism for Enhancing Photocatalytic Stability

**Figure 5:**
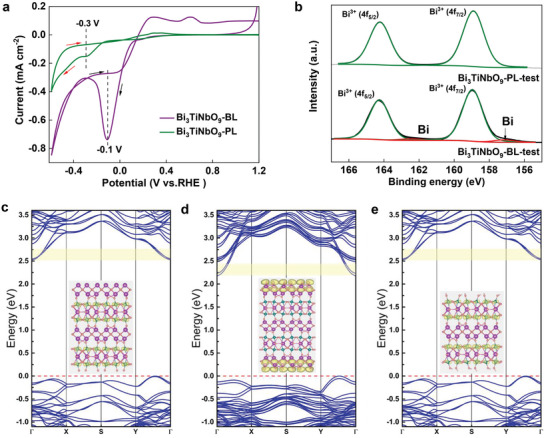
Compound stability analysis. a) Cyclic voltammetry curves of Bi_3_TiNbO_9_‐BL and Bi_3_TiNbO_9_‐PL (0.2 m sodium sulfate aqueous solution, pH = 7). b) X‐ray photoelectron spectroscopy spectra of Bi for Bi_3_TiNbO_9_‐BL and Bi_3_TiNbO_9_‐PL after photochemical reduction test. Calculated band structures of c) Bi_3_TiNbO_9_ bulk, d) Bi_3_TiNbO_9_‐BL, and e) Bi_3_TiNbO_9_‐PL. The red dashed line denotes the Fermi level that was set to 0 eV. The yellow rectangles are used to highlight the energy range for the calculation of band decomposed charge density of CBM, the corresponding atomic structures and the charge density plots with an isosurface level of 0.002 e Å^−3^ are shown as the inserted pictures.

**Figure** **5**a shows the cyclic voltammetry curves of Bi_3_TiNbO_9_‐BL and Bi_3_TiNbO_9_‐PL in a 0.2 m Na_2_SO_4_ aqueous solution, exhibiting distinct electrochemical behaviors under applied cathodic bias. Specifically, typical cathodic peaks associated with Bi^3+^ reduction into metallic Bi^[^ ^12a^ ^]^ appear at −0.1 and −0.3 V versus RHE for Bi_3_TiNbO_9_‐BL and Bi_3_TiNbO_9_‐PL, respectively. Apart from the negative shift of such a peak, the intensity decreased significantly. These two changes indicate that it is much more difficult to reduce Bi^3+^ in Bi_3_TiNbO_9_‐PL than in Bi_3_TiNbO_9_‐BL. The retardance to reduce Bi^3+^ in Bi_3_TiNbO_9_‐PL compared to Bi_3_TiNbO_9_‐BL is also verified by photochemically irradiated treatments, where two samples in deionized water for 5 h were irradiated with a 300 W xenon lamp. Comparison of UV‐vis absorption spectra of two couples of samples (Figure S16, Supporting Information) before and after photochemical reduction suggests that absorption in the range of 400–700 nm is significantly enhanced for Bi_3_TiNbO_9_‐BL due to heavy precipitation of metallic Bi formed from photocorrosion. Such differences between Bi_3_TiNbO_9_‐BL and Bi_3_TiNbO_9_‐PL can be understood by comparing their XPS spectra (Figure 5b and Figure S17, Supporting Information). Compared to Bi_3_TiNbO_9_‐PL‐test, Bi_3_TiNbO_9_‐BL‐test has additional peaks of 157.15 and 162.45 eV, being assignable to metallic Bi.^[^ ^21^ ^]^ Meanwhile, the Nb 3d_3/2_ peak of Bi_3_TiNbO_9_‐BL shifts from 209.05 to 209.27 eV after photochemical reduction, while this peak remains basically unchanged at 209.27 eV for Bi_3_TiNbO_9_‐PL (Figures S6 and S17, Supporting Information). This could be explained as a result that the removal of the surface (Bi_2_O_2_)^2+^ layer during photochemical reduction of Bi_3_TiNbO_9_‐BL causes the exposure of perovskite layer beneath.

On the other hand, density functional theory (DFT) calculation is used to understand the surface electronic structure on the stability of Bi_3_TiNbO_9_. As shown in Figure 5c–e, the conduction band of Bi_3_TiNbO_9_ in the bulk is originated mainly from the perovskite layer, but surface termination significantly affects charge distribution. Specifically, when the surface of Bi_3_TiNbO_9_ is terminated by the (Bi_2_O_2_)^2+^ layer, the CBM has a large contribution from the (Bi_2_O_2_)^2+^ layer, implying that photogenerated electrons tend to accumulate in the (Bi_2_O_2_)^2+^ layer and corrosion easily occurs. This situation changes when Bi_3_TiNbO_9_ is terminated with (BiTiNbO_7_)^2−^ layer. The CBM is now settled in the perovskite layer as to the bulk and is thought to be stable against photocorrosion. Projected density of states (Figures S18–S20, Supporting Information) supported such a difference as well, where the CBM of Bi_3_TiNbO_9_‐BL slab is dominant by Bi 6p orbital, while that of bulk and Bi_3_TiNbO_9_‐PL slab are dominant Ti 3d, Nb 4d orbitals.^[^ ^22^ ^]^

**Figure 6:**
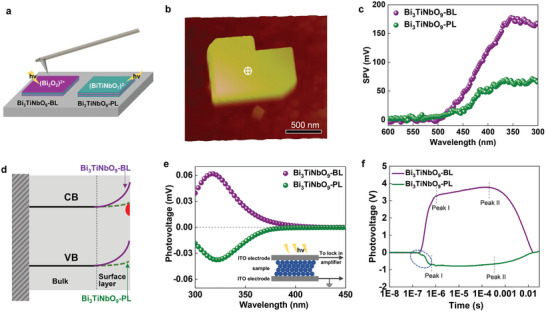
Charge transfer detection. a) Schematic of single particle SPV signal detection. b) Topology image of a single Bi_3_TiNbO_9_ crystal. Spatially resolved SPV spectra c) and the corresponding band bending diagram d) obtained on a single Bi_3_TiNbO_9_‐BL and Bi_3_TiNbO_9_‐PL crystal. SPV spectra e) and transient photovoltage (TPV) response f) of multiple Bi_3_TiNbO_9_‐BL and Bi_3_TiNbO_9_‐PL crystals.

The spatially resolved surface photovoltage spectroscopy (SRSPS) was employed to obtain direct evidence of photogenerated charge transfer on {001} facet of a single Bi_3_TiNbO_9_ nanosheet.^[^ ^21^, ^23^ ^]^ **Figure** **6**a shows the single‐particle surface photovoltage (SPV) measurements, in which one spot in the center of the {001} facet is selected for recording SPV signals of Bi_3_TiNbO_9_‐BL nanosheet or Bi_3_TiNbO_9_‐PL nanosheet, and the corresponding topology image is shown in Figure 6b. Upon light excitation, positive SPV signals can be observed for both Bi_3_TiNbO_9_‐BL and Bi_3_TiNbO_9_‐PL nanosheets (Figure 6c), which can be attributed to the transfer of photogenerated holes to the surface driven by the built‐in electric field in the n‐type surface space charge region band bent upward. The n‐type character is in line with the Mott–Schottky measurements (Figure S2b, Supporting Information). Positive SPV signals from Bi_3_TiNbO_9_‐PL are weaker than that of Bi_3_TiNbO_9_‐BL, indicating that the degree of upward band bending at the Bi_3_TiNbO_9_‐PL surface is smaller and thus the potential barrier for electron transfer to the surface is reduced (Figure 6d). This is responsible for the higher hydrogen evolution activity and lower oxygen evolution activity of Bi_3_TiNbO_9_‐PL compared to Bi_3_TiNbO_9_‐BL (Figure 4a,b). Given their little difference in bulk doping density (inferred from Mott–Schottky plots), the lower band bending of Bi_3_TiNbO_9_‐PL results from its more positive surface states that compensate for its negative surface (BiTiNbO_7_)^2−^ layer. To give more insight into the surface states, we performed SPV measurements on the Bi_3_TiNbO_9_ nanosheet aggregates, as schematically shown in the inset in Figure 6e.^[^ ^24^ ^]^ Interestingly, the positive signals for Bi_3_TiNbO_9_‐BL and negative signals for Bi_3_TiNbO_9_‐PL are obtained in a consistent optical response range (300–400 nm, Figure 6e). The opposite SPV signals are generated and persist on timescales from microseconds to milliseconds (Figure 6f), which typically arise from diffusion or trapping processes.^[^ ^25^ ^]^ Positive signals from Bi_3_TiNbO_9_‐BL can be interpreted as the diffusion of electrons toward the internal bulk region owing to the photo‐Dember effect.^[^ ^25c^ ^]^ In contrast, the negative signals of Bi_3_TiNbO_9_‐PL imply that electron diffusion is impeded by the trapping of electrons at surface states. These results indicate that electrons can be transferred to the (BiTiNbO_7_)^2−^ layer‐terminated surface through an electron trapping regime and meanwhile the holes can be transferred to the lateral surface through surface built‐in electric fields for Bi_3_TiNbO_9_‐PL, whereas only holes can be transferred to the surface for Bi_3_TiNbO_9_‐BL. Therefore, we conclude that both effective electron transfer via surface trapping and effective hole transfer via surface electric field account for high‐performance photocatalytic overall water splitting for Bi_3_TiNbO_9_‐PL.

Furthermore, the atomic structure and electrostatic potential of the Bi_3_TiNbO_9_/Rh interface were also studied to understand the transfer behavior of photogenerated carriers. As can be seen from Figure S21 in the Supporting Information, a Bi—O—Rh bonding is formed at the interface between Bi_3_TiNbO_9_‐BL and Rh cocatalyst with a binding energy of 4.24 eV, and a Ti(Nb)—O—Rh bonding is formed at the interface between Bi_3_TiNbO_9_‐PL and Rh cocatalyst with a binding energy of 5.49 eV. Compared with Bi_3_TiNbO_9_‐BL, Bi_3_TiNbO_9_‐PL has a larger binding energy at the interface, indicating that Rh cocatalyst supported on Bi_3_TiNbO_9_‐PL is characterized by better stability. Besides, a smaller potential barrier (Δ*V* = 3.40 V) was observed at Bi_3_TiNbO_9_‐PL/Rh interface than that at Bi_3_TiNbO_9_‐BL/Rh interface by 1.02 V, resulting in more efficient interfacial electron transfer. Therefore, Bi_3_TiNbO_9_‐PL is more conducive to electron migration to the surface and transfer to the cocatalyst, ensuring the stability of the material in the photocatalytic water‐splitting process.

## 3 Conclusion

In summary, Bi_3_TiNbO_9_, as a typical Aurivillius compound, has the ability to photocatalytic overall water splitting with the assistance of Rh/Cr_2_O_3_ cocatalyst. The surface terminal layer can be selectively regulated by acid etching strategy, as confirmed by TOF‐SIMS analysis, HS‐LEIS spectroscopy, and HAADF‐STEM image. Experimental and theoretical calculations indicate that Bi_3_TiNbO_9_ with (BiTiNbO_7_)^2−^ layer termination is more resistant to photocorrosion in which Bi^3+^ ion is reduced to metallic Bi. Both effective electron transfer via surface trapping and effective hole transfer via surface electric field account for the surface spatial separation of photogenerated electrons and holes in Bi_3_TiNbO_9_‐PL. Moreover, Bi_3_TiNbO_9_‐PL has a smaller Schottky barrier when contacting with the cocatalyst and facilitates the transfer of electrons to the cocatalyst, achieving efficient and stable photocatalytic overall water splitting. This surface modification strategy by simple acid etching can be well extended to other Aurivillius compounds (SrBi_2_Nb_2_O_9_, Bi_4_Ti_3_O_12_, and SrBi_4_Ti_4_O_15_), providing useful guidance for the design of highly active and stable photocatalytic water‐splitting materials.

## Conflict of Interest

The authors declare no conflict of interest.

## Author Contributions

J.H. and Y.K. contributed equally to this work. G.L. led the project. Under the guide of G.L., J.H. together with Y.K. designed and performed the experiments. J.H. drafted the manuscript. J.L. and L.Y. performed the DFT calculations. R.C. and F.F. performed the SRSPS tests. T.X. performed the SPV and TPV tests. Z.L. and H.T. performed the HAADF‐STEM tests. X.X., L.W., and G.L. edited the manuscript. All authors approved the final manuscript.

## Supporting information

Supporting Information

## Data Availability

The data that support the findings of this study are available in the Supporting Information of this article.
