# Supplementary material for: Selective Exposure of Robust Perovskite Layer of Aurivillius‐Type Compounds for Stable Photocatalytic Overall Water Splitting
Source: Adv Sci (Weinh). 2023 May 31;10(23):2302206. doi: 10.1002/advs.202302206 (PMC10427399; doi:10.1002/advs.202302206)
Supplement: Supplementary file 1 — Supporting Information [file ADVS-10-2302206-s001.pdf]

ADVANCED SCIENCE
Open Access

Supporting Information

for *Adv. Sci.*, DOI 10.1002/advs.202302206

Selective Exposure of Robust Perovskite Layer of Aurivillius-Type Compounds for Stable Photocatalytic Overall Water Splitting

*Jie Huang, Yuyang Kang, Jian-An Liu, Ruotian Chen, Tengfeng Xie, Zhongran Liu, Xiaoxiang Xu, He Tian, Lichang Yin, Fengtao Fan, Lianzhou Wang and Gang Liu**

Supporting Information

Selective Exposure of Robust Perovskite Layer of Aurivillius-Type Compounds for Stable Photocatalytic Overall Water Splitting

*Jie Huang, Yuyang Kang, Jian-An Liu, Ruotian Chen, Tengfeng Xie, Zhongran Liu, Xiaoxiang Xu, He Tian, Lichang Yin, Fengtao Fan, Lianzhou Wang, Gang Liu**

J. Huang, Y. Kang, J. Liu, L. Yin, G. Liu
Shenyang National Laboratory for Materials Science, Institute of Metal Research, Chinese Academy of Sciences, 72 Wenhua Road, Shenyang 110016, China.
E-mail: gangliu@imr.ac.cn
J. Huang, J. Liu, L. Yin, G. Liu
School of Materials Science and Engineering, University of Science and Technology of China, 72 Wenhua Road, Shenyang 110016, China.
R. Chen, F. Fan
State Key Laboratory of Catalysis, Dalian National Laboratory for Clean Energy, iChEM, Dalian Institute of Chemical Physics, Chinese Academy of Sciences, Dalian 116023, China
T. Xie
College of Chemistry, Jilin University, Changchun, 130012, China.
Z. Liu, H. Tian
Center of Electron Microscopy, School of Materials Science and Engineering, Zhejiang University, Hangzhou, 310027, China.
X. Xu
School of Chemical Science and Engineering, Tongji University, Shanghai, 200092, China.
L. Wang
Nanomaterials Centre, School of Chemical Engineering and Australian Institute for Bioengineering and Nanotechnology, The University of Queensland, QLD 4072, Australia.

Experimental Section

Synthesis of Bi_3TiNbO_9-BL nanosheets. Bi_2O_3 (0.699 g, Sinopharm (China)), anatase TiO_2 (0.08 g, Aldrich, 99%), Nb_2O_5 (0.133 g, Aladdin, 99.9%) and NaCl-KCl (8 g, Sinopharm (China)) mixed molten salt were mixed up and ground with mortar and pestle for half an hour. The resulting mixtures were transferred to an alumina crucible and calcined in a muffle furnace. The calcination was set at 800 ºC with a ramping rate of 5 ºC min^{-1} and a duration of 2 hours. The final products were filtered with hot deionized water to completely remove the flux agents and then dried in air.

Synthesis of Bi_3TiNbO_9-PL nanosheets. Bi_3TiNbO_9-BL nanosheets (250 mg) were dispersed in a hydrochloric acid solution (125 mL, 0.32 mol L^{-1}) under continuous stirring for 5 h. The sample was then separated by filtration and rinsed with a large amount of deionized water until the supernatant was neutral.

Cocatalyst deposition. Sample powders (150 mg) were dispersed in a photoreactor containing aqueous methanol solution (100 mL, 10 vol%) and rhodium chloride (0.9 mL, 1 mg mL^{-1}, $RhCl_3$, Alfa, 99.9%) solution. After evacuation, the suspension was irradiated under a full spectrum xenon lamp for half an hour. Subsequently, potassium chromate (0.9 mL, 1mg mL^{-1}, K_2CrO_4, Sigma-Aldrich, 99%) solution was added to the reactor. After evacuation, the suspension was irradiated again under the same conditions for one hour. The final product was filtrated, washed thoroughly with distilled water and dried in air.

Photocatalytic activity evolution. Photocatalytic overall water splitting was conducted in an air-tight automatic test system (Beijing Perfectlight Technology Co., Ltd., Labsolar-6A) equipped with a 250 mL photoreactor. The specific operation process was as follows: 50 mg sample powders loaded with cocatalyst were dispersed into the photoreactor containing 100 mL deionized water. After evacuation to remove dissolved air, the photoreactor was irradiated with a 300 W xenon lamp (Beijing Perfectlight Technology Co., Ltd., PLS-SXE300D). Gas evolution within the photoreactor was analyzed by gas chromatography (Agilent Technologies, 6890N).

Materials characterization. X-ray diffraction patterns of the samples were collected on a Rigaku D/max 2400 spectrometer using Cu Kα X-rays of wavelength 1.54056 Å. The morphology of the samples was investigated by Scanning Electron Microscope (Nova NanoSEM430). The contact angle test was conducted under the contact angle measuring instrument (Shanghai Zhongchen Digital Technology Apparatus Co., Ltd. JC2000D1). Diffusion reflection absorption spectra were obtained using a spectrophotometer (Jasco V-770) equipped with an integrated sphere in diffusion reflection mode. The compositions and

chemical states of the samples were analyzed using X-ray photoelectron spectroscopy (Thermo Escalab 250, a monochromatic Al Kα X-ray source). All binding energies were referenced to the C 1s peak (284.6 eV) that arises from adventitious carbon. Cyclic voltammetry curves were performed using BioLogic multichannel electrochemical workstation. The test procedure is as follows: (1) 20 mg sample powders and a slight amount of iodine were dispersed into 25 mL acetone; (2) ultrasound is then performed to disperse the sample powders; (3) constant voltage mode is adopted to electrophoretic the sample powders onto FTO (10 V, 5 min). Pt electrode and saturated Ag/AgCl electrode were used as counter electrode and reference electrode, respectively. 0.2 M Na_2SO_4 was selected as the electrolyte for cyclic voltammetry curves test.

Spatially Resolved Surface Photovoltage Spectroscopy (SRSPS) measurement. The SRSPS setup was installed in a KPFM setup consisting of a modified Dimension Icon and operated under an ambient atmosphere, Platinum/iridium-coated silicon tips with a spring constant of 1–5 N m^{-1} and a resonance frequency of 60–100 kHz (Bruker SCM-PIT) was used. The surface potential signals were first mapped in amplitude modulated (AM-KPFM) mode at an a.c. voltage of 0.5 V and a tip lift height of 50 nm. The difference between the signal under dark state and illumination (500 W Xe lamp, light intensity: 2 mW cm^{-2}) at the same position location was extracted as surface photovoltaic spectra.

Surface Photovoltage (SPV) measurement. The system included a lock-in amplifier (SR830, Stanford Research Systems, Inc.) with a light chopper (SR540), a 500 W xenon lamp with a monochromator (SBP500, Zolix) as the light source, and a photovoltaic cell. The monochromatic light chopped with a frequency of 23 Hz is focused into the photovoltaic cell. The photovoltaic signal is amplified by a lock-in amplifier and transmitted to a computer for recording.

Transient Photovoltage (TPV) measurement. The system included a Nd: YAG laser source (Q-smart 450 Quantel) providing laser radiation pulse (wavelength: 355 nm, pulse width: 5 ns and Intensity: 100 μJ), a 500 MHz digital phosphor oscilloscope (TDS 5054, Tektronix) with a preamplifier (5003 Brookdeal Electronics), and a parallel-plate capacitor-like sample chamber.

Calculation detail. Density functional theory (DFT) calculations were performed using the Vienna Ab-initio Simulation Package[1] with the projector augmented wave method[2]. Perdew-Burke-Ernzerhof (PBE) functional[3] for the exchange-correlation (XC) term was used for all calculations. The projector augmented wave method (PAW) was used at a plane-wave cutoff of 500 eV to describe the electron-ion interaction. The valence electron configurations were Bi: $5d^{10}6s^26p^3$, Ti: $3s^23p^63d^24s^2$, Nb: $4s^24p^64d^45s^1$, O: $2s^22p^4$, Rh: $4p^64d^85s^1$.

The calculated lattice constant for orthorhombic Bi_3TiNbO_9 (space group $A2_1am$) are

a=5.534 Å, b=5.459 Å, c=25.578 Å, in well agreement with reported experimental work[4] (a=5.43 Å, b=5.39 Å, c=25.05 Å). The relevant surface and interface structure were simply constructed using a 1x1 slab model. For Bi_3TiNbO_9-BL, a Bi_3TiNbO_9 (001) slab model is composed of three $(Bi_2O_2)^{2+}$ layers and two perovskite layers, while the Bi_3TiNbO_9-PL slab is composed of one $(Bi_2O_2)^{2+}$ layer and two perovskite layers. To discuss the stability of the supported co-catalyst (Rh), a six-atomic-layer Rh (001) slab was combined with Bi_3TiNbO_9-BL and Bi_3TiNbO_9-PL slabs to construct Rh/Bi_3TiNbO_9 heterojunctions (as shown in Figure S18).

All geometries were fully relaxed with convergence criteria of 1×10^{-6} eV in total energy and 0.02 eV $Å^{-1}$ in residual force. For all slabs, the vacuum thickness was set to be 20 Å along the z direction. Gamma-centered Monkhorst-Pack grids with 7x7x2 and 5x5x1 k-point sampling were used for bulk Bi_3TiNbO_9 and relevant slabs, respectively.

In order to evaluate the stability of the supported Rh co-catalyst on Bi_3TiNbO_9-BL and Bi_3TiNbO_9-PL, the binding energy (ΔE) was calculated using the following formula,

$$\Delta E = E_{Rh/BTNO} - E_{BTNO} - E_{Rh}$$

Here, the $E_{Rh/BTNO}$, E_{BTNO}, and E_{Rh} is the total energy of Rh/Bi_3TiNbO_9 heterojunction, Bi_3TiNbO_9-BL or Bi_3TiNbO_9-PL slab, and Rh (001) slab, respectively. Additionally, the electrostatic potentials of two relevant interfaces were calculated to discuss the difficulty of electron transfer from Bi_3TiNbO_9 to Rh *via* Bi_3TiNbO_9 (001)/Rh (001) interfaces, such an approach has been successfully used to evaluate the interfacial charge transfer of $PbTiO_3/TiO_2$ heterostructure[5] and metal/TiO_2 heterostructure[6].

Apparent quantum efficiency (AQY) measurement. Firstly, the amount of hydrogen produced by photocatalytic overall water splitting and photon density at monochromatic wavelength are obtained, and then calculated by AQY expression.

$$\text{AQY} = \frac{\text{The mount of Hydrogen} \times 2}{\text{Photon Flux/m}^2 \times \text{A} \times \text{t}}$$

where, the amount of hydrogen is the hydrogen obtained by photocatalytic overall water splitting during the time period t (unit, μmol). Photon Flux/m^2 is photons per unit area per unit time (unit, μmol/s/m^2). A is the area through which the photon passes.

Supplementary Figures

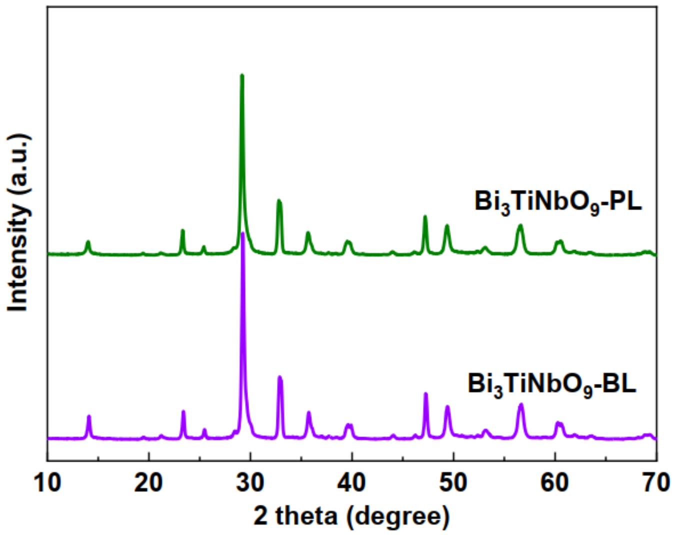

Figure S1. X-ray diffraction patterns of Bi_3TiNbO_9-BL and Bi_3TiNbO_9-PL.

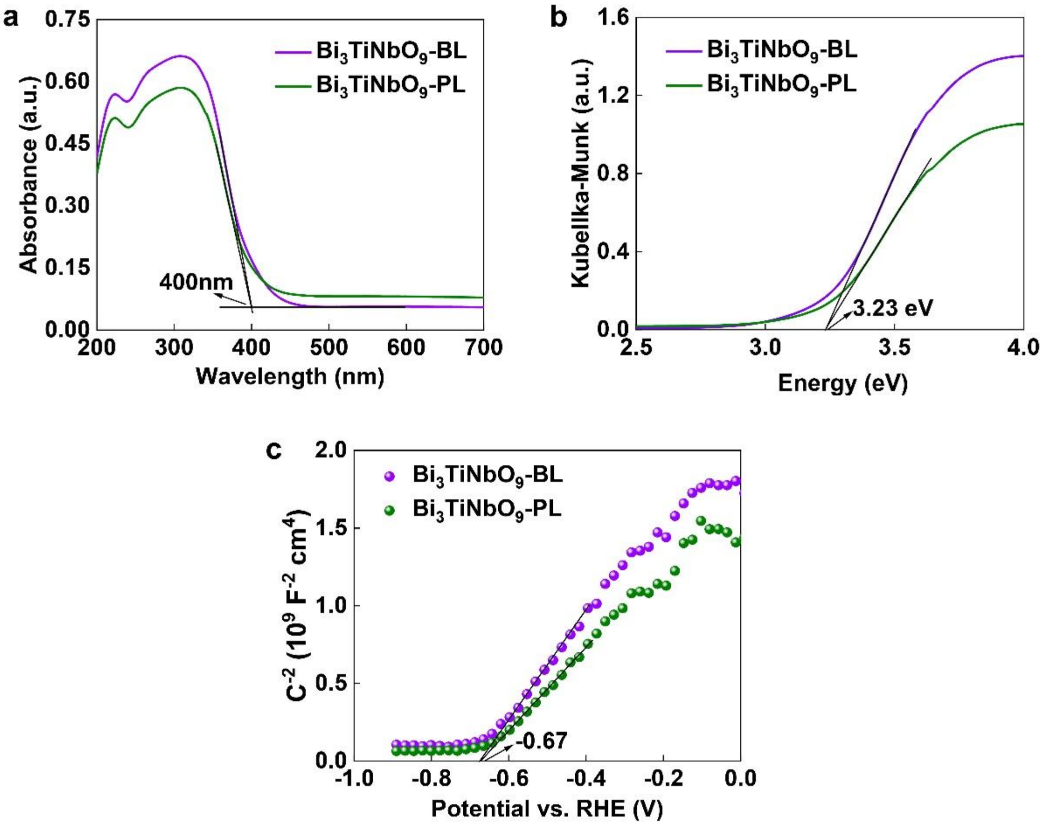

Figure S2. a) UV–vis diffuse reflectance spectra of Bi_3TiNbO_9-BL and Bi_3TiNbO_9-PL. b) Kubellka–Munk transformed diffuse reflectance spectra. c) Mott-Schottky curves.

Figure S2 shows that the band gap of Bi_3TiNbO_9-BL and Bi_3TiNbO_9-PL is 3.23 eV, and the flat band potential is -0.67 V vs. RHE. In addition, the Mott-Schottky curves exhibit a positive slope, indicating that Bi_3TiNbO_9-BL and Bi_3TiNbO_9-PL are n-type semiconductor. With

reference to the position of the conduction band of n-type semiconductor being 0.2 eV higher than the flat band potential, it can be deduced that the conduction band positions of Bi_3TiNbO_9-BL and Bi_3TiNbO_9-PL are both -0.87 V vs. RHE. Subsequently, it is inferred that the valence band position is 2.36 vs. RHE.

Table S1. ICP-OES test results of aqueous solution collected after etching Bi_3TiNbO_9-BL with hydrochloric acid. The solution after water immersion under the same conditions was used as the comparison.

	Bi /mg L^{-1}	Ti /mg L^{-1}	Nb /mg L^{-1}
hydrochloric acid etching-1	158.2	0.79	<0.1
hydrochloric acid etching -2	165.2	-	-
water immersion	<0.1	<0.1	<0.1

The test results of two batches of acid etching solution were consistent, and only Bi^{3+} ions were tested, while the content of Ti^{4+} and Nb^{5+} was negligible. Under the same conditions, Bi_3TiNbO_9-BL was impregnated with deionized water and no ions were detected in the resulting solution.

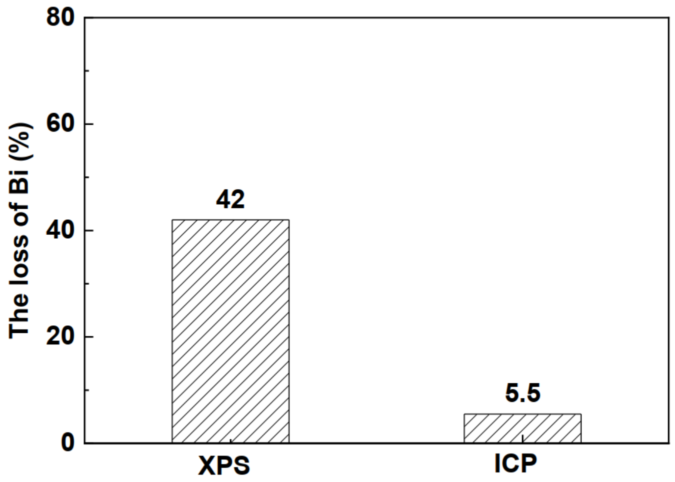

Figure S3. Comparison of the loss percentage of Bi ion of Bi_3TiNbO_9-PL with Bi_3TiNbO_9-BL determined by XPS and ICP methods.

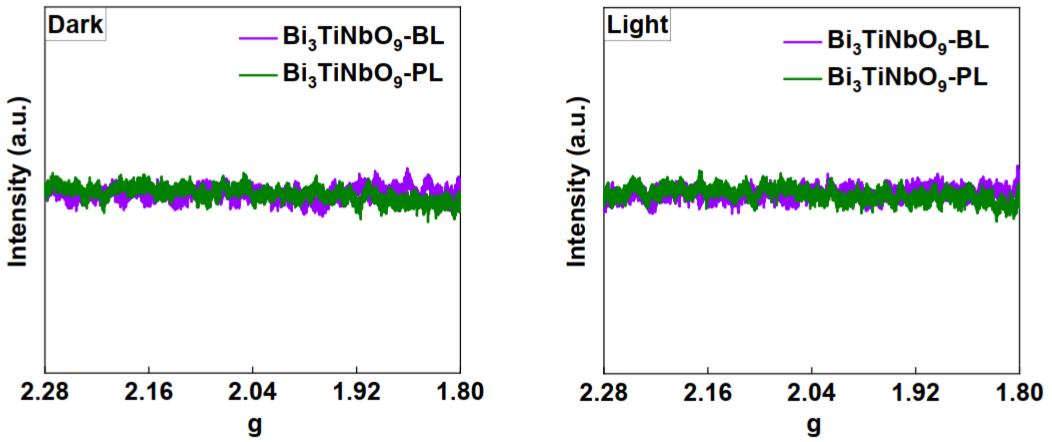

Figure S4. Electron spin resonance (ESR) spectra of Bi_3TiNbO_9-BL and Bi_3TiNbO_9-BL in dark and light irradiation.

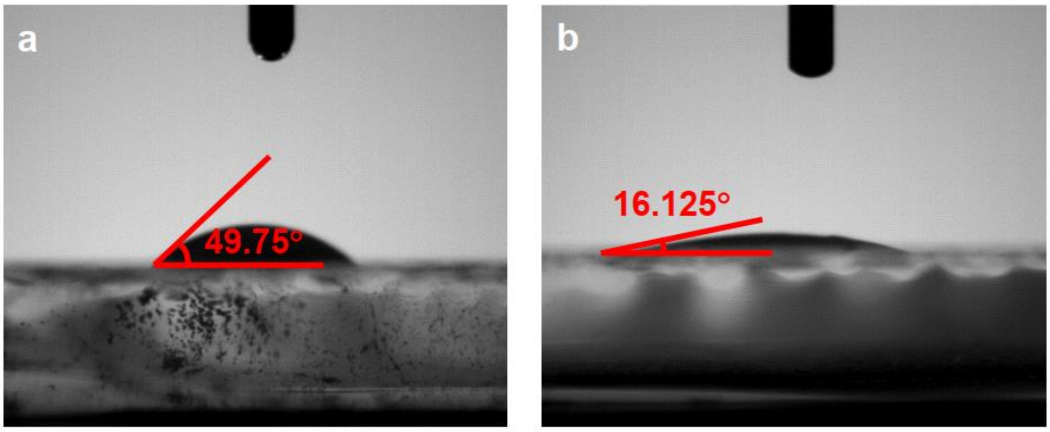

Figure S5. Photographs of water droplet on the sample surface: a) Bi_3TiNbO_9-BL, b) Bi_3TiNbO_9-PL. The external water contact angle (CA) was determined to be 49.75° and 16.125° for Bi_3TiNbO_9-BL and Bi_3TiNbO_9-PL, respectively. CA of Bi_3TiNbO_9 was reduced after the surface structure transition, indicating an improvement in hydrophilic properties.

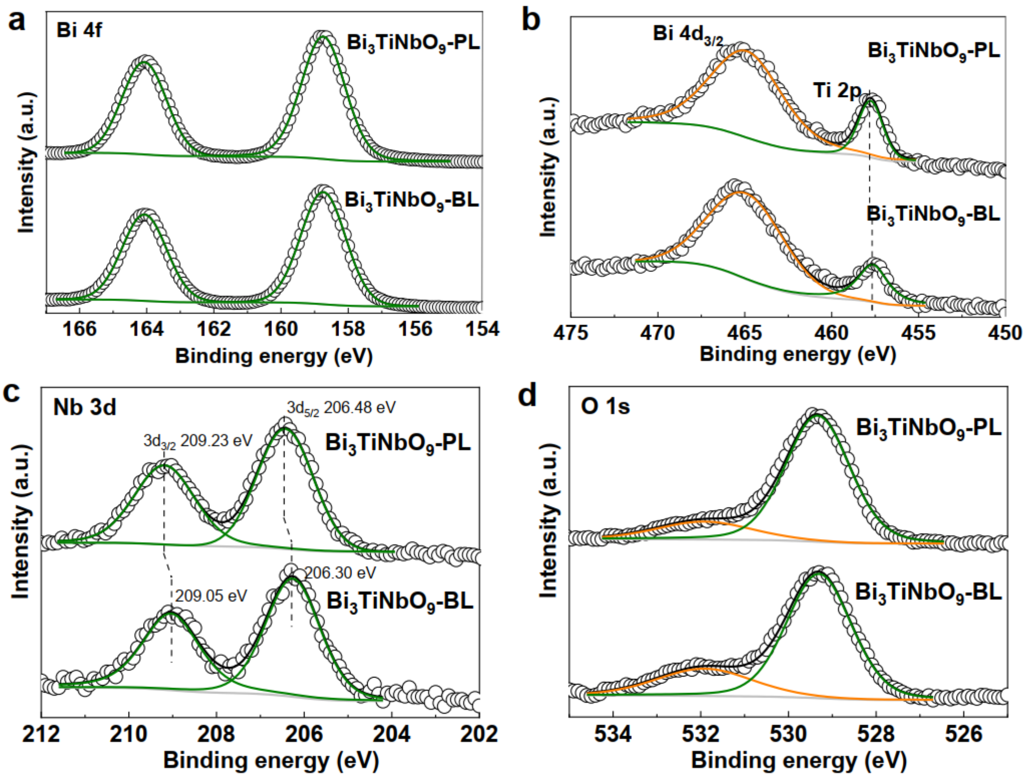

Figure S6. XPS spectra of Bi_3TiNbO_9-BL and Bi_3TiNbO_9-PL: a) Bi 4f, b) Ti 2p + Bi $4d_{3/2}$, c) Nb 3d and d) O 1s.

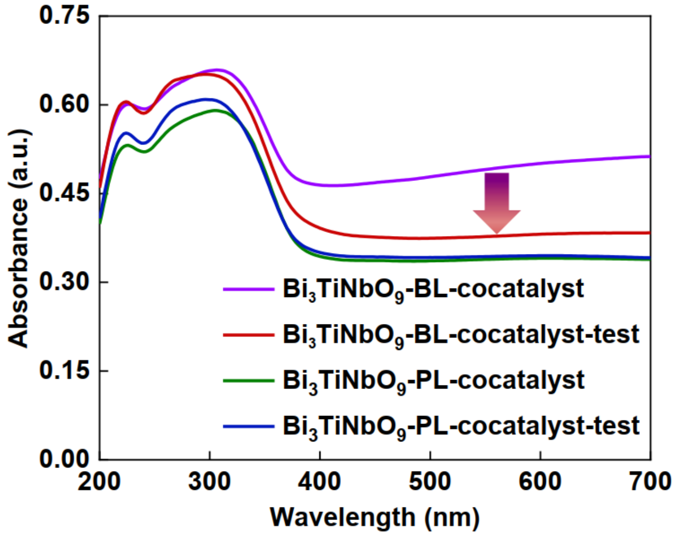

Figure S7. UV–vis diffuse reflectance spectra of cocatalyst modified Bi_3TiNbO_9-BL and Bi_3TiNbO_9-PL before and after the photocatalytic water splitting test.

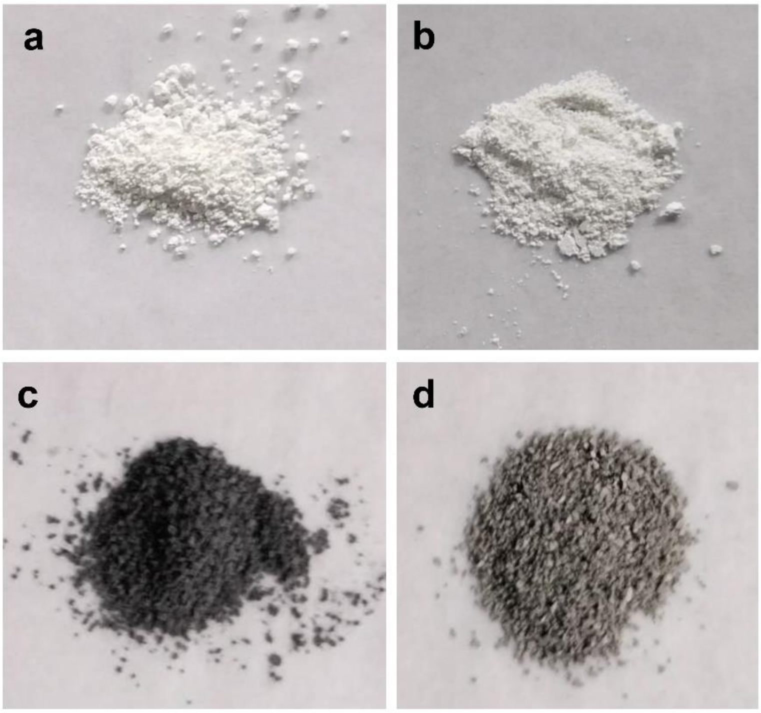

Figure S8. Photographs of powder samples: a) Bi_3TiNbO_9-BL, b) Bi_3TiNbO_9-PL, c) Bi_3TiNbO_9-BL-Rh/Cr_2O_3, d) Bi_3TiNbO_9-PL-Rh/Cr_2O_3.

It is evident that both the Bi_3TiNbO_9-BL and Bi_3TiNbO_9-PL samples appear white in color, but their color changes significantly after cocatalyst deposition. Specifically, Bi_3TiNbO_9-BL-Rh/Cr_2O_3 exhibits a dark gray color, while Bi_3TiNbO_9-PL-Rh/Cr_2O_3 appears light gray.

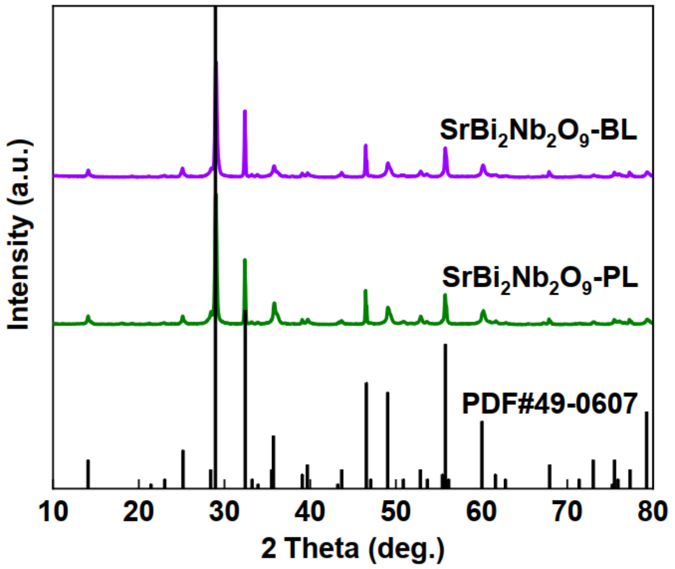

Figure S9. X-ray diffraction patterns of $SrBi_2Nb_2O_9$-BL and $SrBi_2Nb_2O_9$-PL.

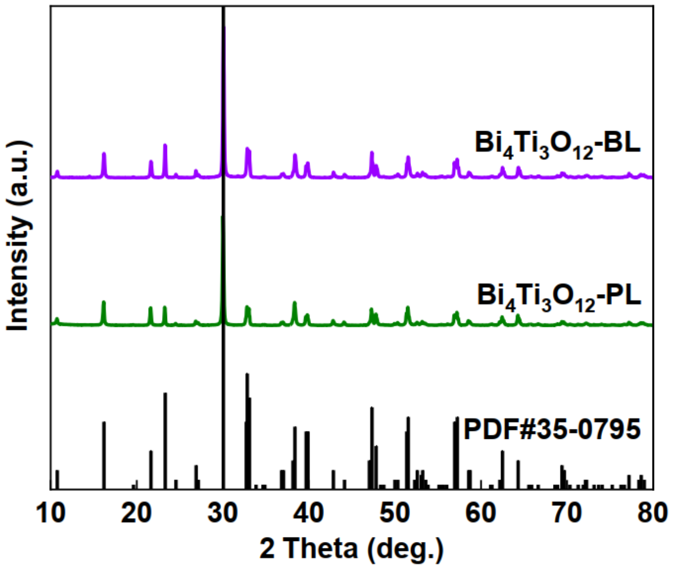

Figure S10. X-ray diffraction patterns of $Bi_4Ti_3O_{12}$-BL and $Bi_4Ti_3O_{12}$-PL.

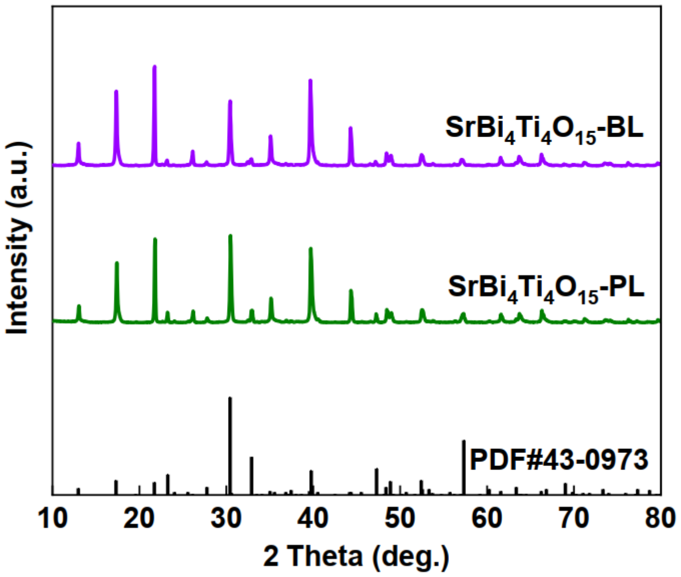

Figure S11. X-ray diffraction patterns of $SrBi_4Ti_4O_{15}$-BL and $SrBi_4Ti_4O_{15}$-PL.

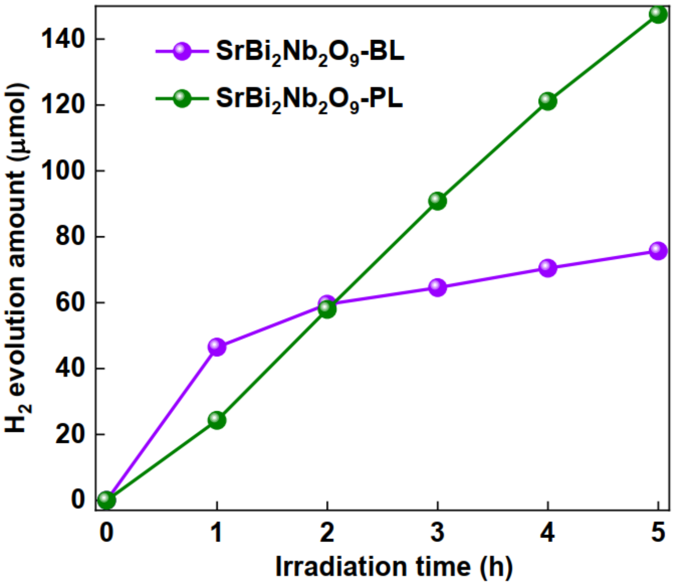

Figure S12. Comparison of photocatalytic hydrogen evolution over $SrBi_2Nb_2O_9$-BL and $SrBi_2Nb_2O_9$-PL photocatalysts. (Reaction conditions: 50 mg photocatalyst, 1 wt% Pt cocatalyst, 10 mL methanol + 90 mL H_2O)

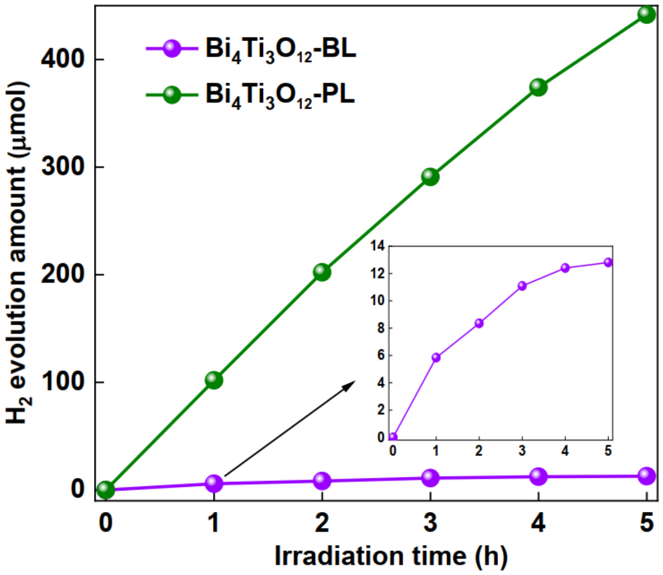

Figure S13. Comparison of photocatalytic hydrogen evolution over $Bi_4Ti_3O_{12}$-BL and $Bi_4Ti_3O_{12}$-PL photocatalysts. (Reaction conditions: 50 mg photocatalyst, 1% Pt, 10 mL methanol + 90 mL H_2O)

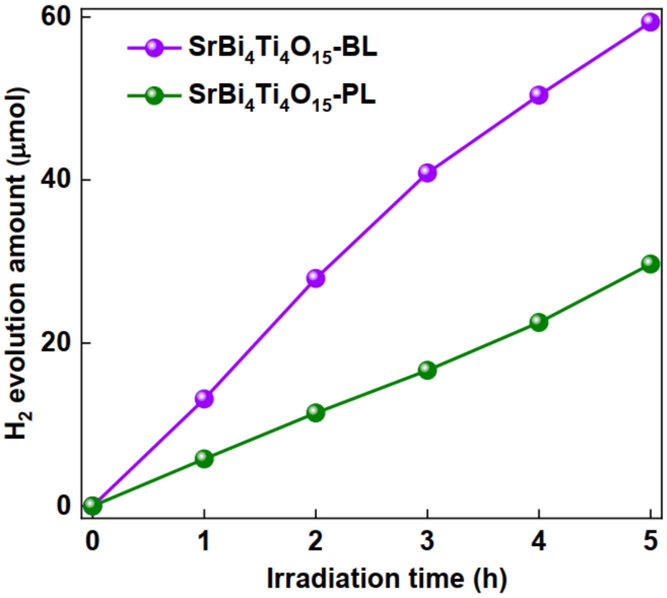

Figure S14. Comparison of photocatalytic hydrogen evolution over $SrBi_4Ti_4O_{15}$-BL and $SrBi_4Ti_4O_{15}$-PL photocatalysts. (Reaction conditions: 50 mg photocatalyst, 1% Pt, 10 mL methanol + 90 mL H_2O)

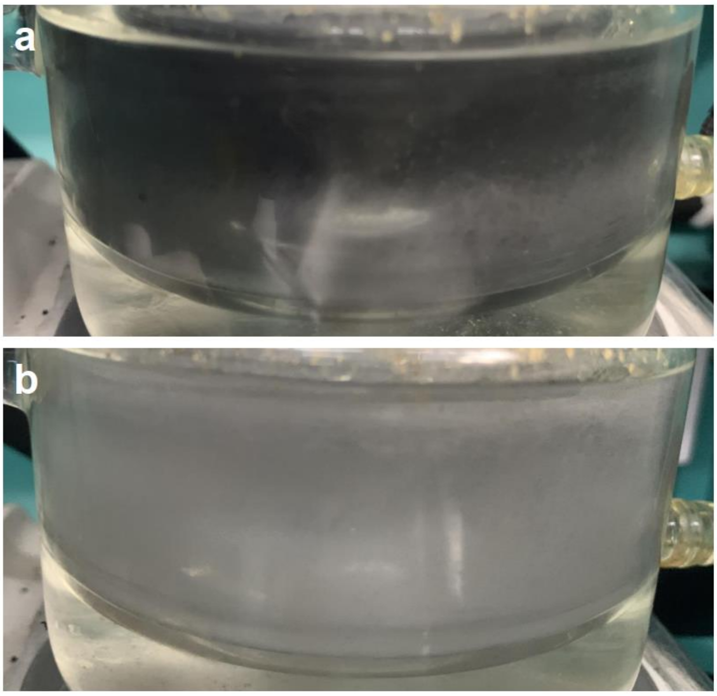

Figure S15. Optical photographs of exposed $(Bi_2O_2)^{2+}$ layer samples a), $SrBi_2Nb_2O_9$-BL, $Bi_4Ti_3O_{12}$-BL, $SrBi_4Ti_4O_{15}$-BL) and perovskite layer exposed samples b), $SrBi_2Nb_2O_9$-PL, $Bi_4Ti_3O_{12}$-PL, $SrBi_4Ti_4O_{15}$-PL) after photocatalytic hydrogen evolution. (Note: The color changes of the three sample groups are consistent after reaction, so only one group is placed.)

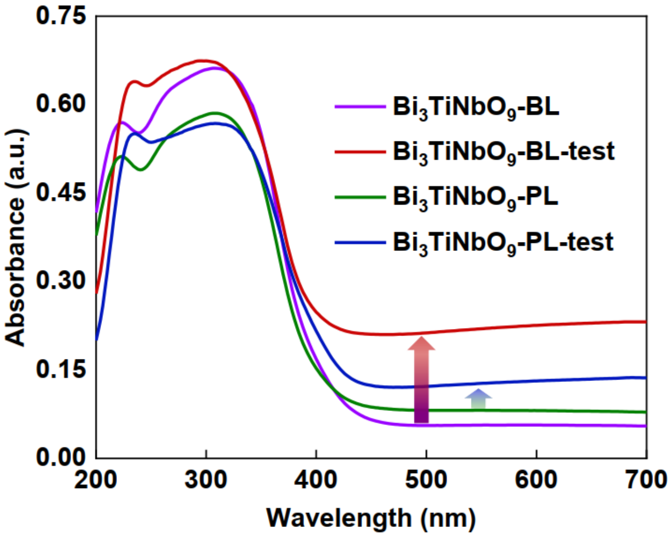

Figure S16. Diffuse reflectance absorption spectra of Bi_3TiNbO_9-BL and Bi_3TiNbO_9-PL before and after photochemical test.

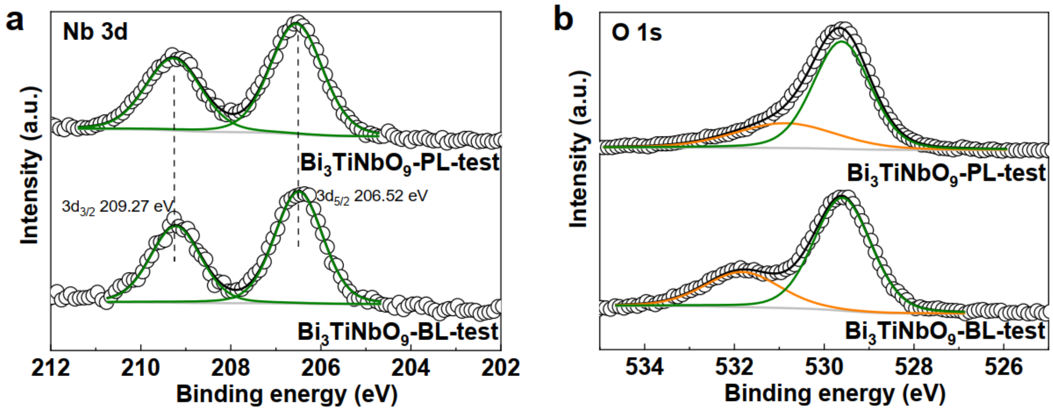

Figure S17. a) Nb 3d and b) O 1s XPS spectra of Bi_3TiNbO_9-BL and Bi_3TiNbO_9-PL after photochemical reduction.

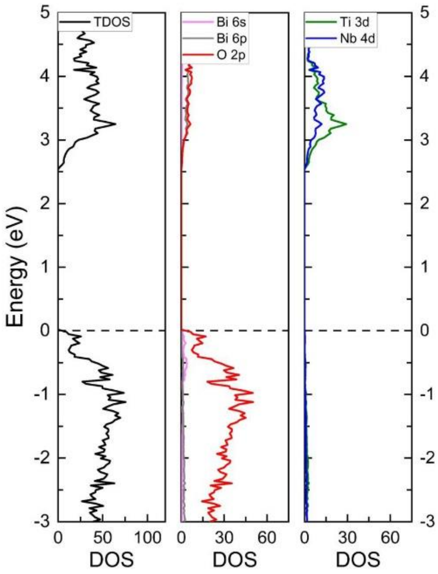

Figure S18. Calculated projected density of states of bulk phase of Bi_3TiNbO_9. The dashed black lines denote the fermi energy that is set to 0.

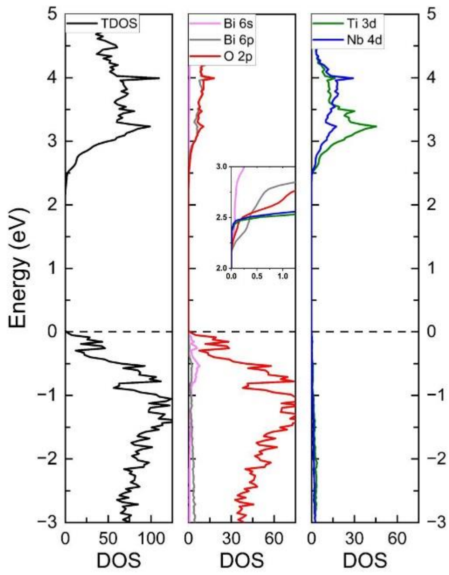

Figure S19. Calculated projected density of states of Bi_3TiNbO_9-BL. The dashed black lines denote the fermi energy that is set to 0.

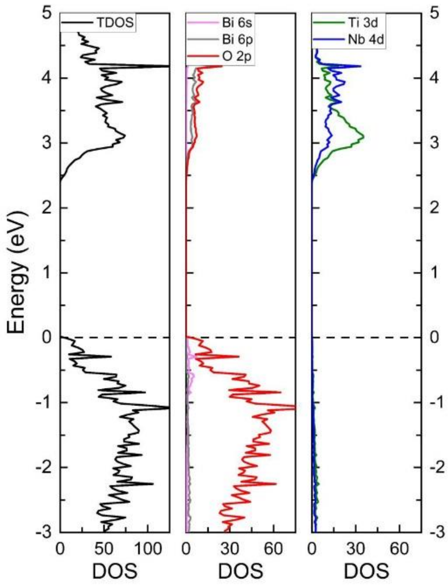

Figure S20. Calculated projected density of states of Bi_3TiNbO_9-PL. The dashed black lines denote the fermi energy that is set to 0.

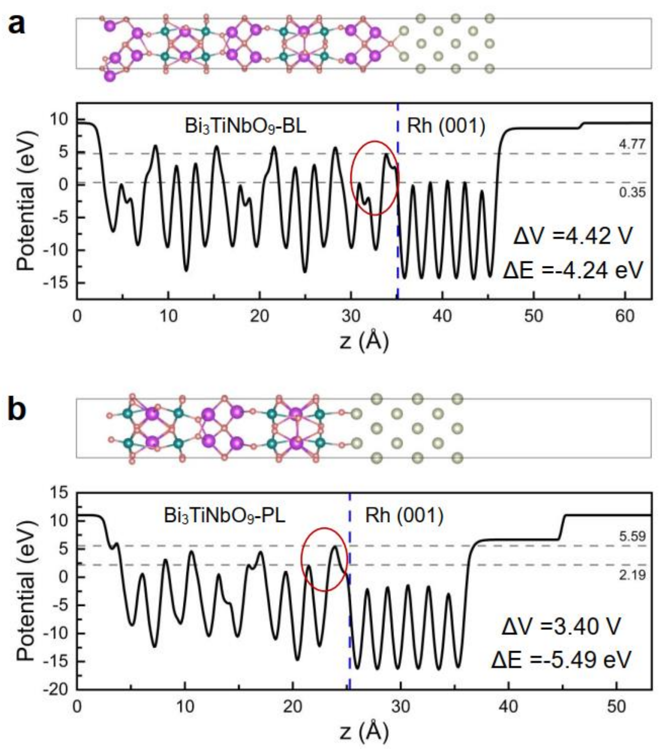

Figure S21. Fully relaxed atomic structures of two Rh/Bi_3TiNbO_9 heterojunctions and the plane averaged electrostatic potential as a function of the interlayer distance along the *z* direction. a) Bi_3TiNbO_9-BL /Rh (001) interfaces. b) Bi_3TiNbO_9-PL /Rh (001) interfaces.

References

[1] a) G. Kresse, J. Hafner, *Phys. Rev. B* **1993**, *47*, 558; b) G. Kresse, J. Hafner, *Phys. Rev. B* **1994**, *49*, 14251; c) G. Kresse, J. Furthmüller, *Comput. Mater. Sci.* **1996**, *6*, 15; d) G. Kresse, J. Furthmüller, *Phys. Rev. B* **1996**, *54*, 11169.

[2] a) P. E. Blöchl, *Phys. Rev. B* **1994**, *50*, 17953; b) G. Kresse, D. Joubert, *Phys. Rev. B* **1999**, *59*, 1758.

[3] a) J. P. Perdew, K. Burke, M. Ernzerhof, *Phys. Rev. Lett.* **1996**, *77*, 3865; b) J. P. Perdew, K. Burke, M. Ernzerhof, *Phys. Rev. Lett.* **1997**, *78*, 1396.

[4] H. Yu, F. Chen, X. Li, H. Huang, Q. Zhang, S. Su, K. Wang, E. Mao, B. Mei, G. Mul, T. Ma, Y. Zhang, *Nat. Commun.* **2021**, *12*, 4594.

[5] G. Liu, L.-C. Yin, J. Pan, F. Li, L. Wen, C. Zhen, H.-M. Cheng, *Adv. Mater.* **2015**, *27*, 3507.

[6] G. Liu, L. Ma, L.-C. Yin, G. Wan, H. Zhu, C. Zhen, Y. Yang, Y. Liang, J. Tan, H.-M. Cheng, *Joule* **2018**, *2*, 1095.
